# Supplementary material for: A Novel Intragenic Duplication in the HDAC8 Gene Underlying a Case of Cornelia de Lange Syndrome
Source: Genes (Basel). 2022 Aug 8;13(8):1413. doi: 10.3390/genes13081413 (PMC9408140; doi:10.3390/genes13081413)
Supplement: Supplementary file 1 [file genes-13-01413-s001.zip › genes-1846460-supplementary.pdf]

Table S1. Insertion 96 pb sequence

Insertion 96 pb
AATATGCCTGTGAACACCTAGAGGACCCCAGAGGAGGTCTCATCCATCTTTATAGTCCCTC TGCAACAAGCACAATGACCTGCATGTGATGTGCAG

[1]Tables may have a footer.

Table S2. WT protein sequence

WT HDAC8 sequence
MEEPEEPADSGQSLVPVYIYSPEYVSMCDSLAKIPKRASMVHSLIEAYALHKQMRIVKPKVASME EMATFHTDAYLQHLQKVSQEGDDDHPDSIEYGLGYDCPATEGIFDYAAAIGGATITAAQCLID GMCKVAINWSGGWHHAKKDEASGFCYLNDAVLGILRLRRKFERILYVDLDLHHGDGVEDAFS FTSKVMTVSLHKFSPGFFPGTGDVSDVGLGKGRYYSVNVPIQDGIQDEKYYQICESVLKEVYQAF NPKAVVLQLGADTIAGDPMCSFNMTPVGIGKCLKYILQWQLATLILGGGGYNLANTARCWTY LTGVILGKTLSSEIPDHEFFTAYGPDYVLEITPSCRPDRNEPHRIQQILNYIKGNLKHVV

Table S3. Variant protein sequence

Variant protein HDAC8 sequence
MEEPEEPADSGQSLVPVYIYSPEYVSMCDSLAKIPKRASMVHSLIEAYALHKQMRIVKPKVASME EMATFHTDAYLQHLQKVSQEGDDDHPDSIEYGLGYDCPATEGIFDYAAAIGGATITAAQCLID GMCKVAINWSGGWHHAKKDEASGFCYLNDAVLGILRLRRKFERILYVDLDLHHGDGVEDAFS FTSKVMTVSLHKFSPGFFPGTGDVSDVGLGKGRYYSVNVPIQDGIQDEKYYQICESVLKEVYQAF NPKAVVLQLGADTIAGDPMCSFNMTPVGIGKCLKYILQWQLATLILGGGGYNLANTARCWTY LTGVILGKTLSSEIPDHENMPVNT*RTPEEVSSIFIVPLQQAQ*PACDVQFFTAYGPDYVLEITPSCR PDRNEPHRIQQILNYIKGNLKHVV
